# Pediatric NMOSD: A Review and Position Statement on Approach to Work-Up and Diagnosis

**DOI:** 10.3389/fped.2020.00339

**Published:** 2020-06-25

**Authors:** Silvia Tenembaum, E. Ann Yeh, The Guthy-Jackson Foundation International Clinical Consortium (GJCF-ICC), Hesham Abboud

**Affiliations:** ^1^Department of Neurology, National Pediatric Hospital Dr. J. Garrahan, Buenos Aires, Argentina; ^2^Division of Neurology, Department of Pediatrics, SickKids Research Institute, The Hospital for Sick Children, University of Toronto, Toronto, ON, Canada

**Keywords:** pediatric, neuroinflammation, NMOSD, MOG, treatment, diagnosis

## Abstract

Neuromyelitis Optica Spectrum Disorder (NMOSD) is an inflammatory demyelinating disease of the central nervous system (CNS) primarily affecting the optic nerves and spinal cord, but also involving other regions of the CNS including the area postrema, periaqueductal gray matter, and hypothalamus. Knowledge related to pediatric manifestations of NMOSD has grown in recent years, particularly in light of newer information regarding the importance of not only antibodies to aquaporin 4 (AQP4-IgG) but also myelin oligodendrocyte glycoprotein (MOG-IgG) in children manifesting clinically with this syndrome. In this review, we describe the current state of the knowledge related to clinical manifestations, diagnosis, and chronic therapies for children with NMOSD, with emphasis on literature that has been published in the last 5 years. Following the review, we propose recommendations for the assessment/follow up clinical care, and treatment of this population.

## Introduction

Neuromyelitis Optica Spectrum Disorder (NMOSD) is a chronic relapsing condition characterized by inflammatory episodes of the central nervous system, primarily affecting the optic nerves and spinal cord, but also involving other regions of the central nervous system including the area postrema, periaqueductal gray matter, and hypothalamus. Specific cases may also show a variety of abnormalities in the brain, involving the periependymal and subcortical white matter, the corpus callosum, corticospinal tracts and deep gray matter. Since the landmark description of a highly sensitive and specific antibody against the AQP4 channel—AQP4-IgG antibody—in 2004 (1), knowledge about this syndrome and its treatment has increased substantially.

While much work has focused on manifestations of the syndrome in the adult population, recent years have seen an exponential rise in knowledge regarding NMOSD in children. This has occurred together with the establishment of the clinical utility/relevance of another antibody that appears with great frequency in children with neuroinflammatory disorders, and which has been associated with a similar clinical syndrome, albeit with specific and distinct manifestations, antibodies to myelin oligodendrocyte glycoprotein (MOG) (2, 3).

In this review, we describe the current state of the knowledge related to clinical manifestations, diagnosis, and chronic therapies for children with NMOSD, with emphasis on literature that has been published in the last 5 years. We will not discuss treatment of acute relapses in this review. Following the review, we propose recommendations for the assessment/follow up clinical care, and treatment of this population.

## Review of Current Knowledge

### Definitions

Diagnostic criteria for neuromyelitis optica (NMO) have evolved since the initial 1999 publication which described NMO as an inflammatory CNS syndrome distinct from MS. Initial criteria required the presence of optic neuritis, longitudinally extensive transverse myelitis, CSF pleocytosis >50 cells/mm^3^, and the absence of brain MRI lesions or absence of lesions typical for MS (4). Since 1999, the criteria have undergone 2 more revisions. In 2006, the criteria saw the introduction of AQP4-IgG -positivity as a supportive criterion and loosening of the supportive criterion related to—*brain MRI abnormalities not meeting MS diagnostic criteria*. The diagnostic entity continued to be called NMO. Over the next decade, identification of the antibody and widespread availability of testing made it clear that a spectrum of clinical and MRI manifestations outside of optic neuritis and transverse myelitis should be included in this clinical entity, including specific focal abnormalities such as area postrema and hypothalamic involvement that might occur *in the absence of* optic nerve or spinal cord involvement. This led to a nosological change, to the current consensus criteria based entity of *Neuromyelitis Optica Spectrum Disorder* (NMOSD) (5). Core criteria include optic neuritis, acute myelitis, area postrema syndrome, acute brainstem syndrome, narcolepsy/acute diencephalic syndrome, and symptomatic cerebral syndrome with NMOSD-typical brain lesions. Importantly, for diagnosis, only one core criterion is necessary in the presence of AQP4-antibody. The criteria for making the diagnosis in seronegative patients or in the absence of access to AQP4-IgG testing included dissemination in space with the presence of two core clinical criteria and additional MRI requirements. The diagnosis is contingent on the absence of alternate reasons explaining the syndrome. These diagnostic criteria have been validated in the pediatric age group (6).

### Demographics and Epidemiology

Previous epidemiologic studies have focused primarily on ***AQP4-IgG related NMOSD***, and have revealed that NMOSD prevalence varies by race, being higher in non-white than in white populations (7, 8). A study conducted in Australia and New Zealand showed that NMOSD was three times more prevalent in individuals with Asian ancestry than in the remaining population with predominantly European ancestry (9). In addition, the NMOSD/multiple sclerosis ratio has been reported to be higher in Asia than in Western countries (10). A recent study using data from a large international cohort of adult patients with NMOSD revealed that race affected the clinical phenotype, age at onset, and severity of attacks, but the overall clinical outcome was most dependent on early and effective immunosuppressive treatment initiation (11).

Although infrequent, NMOSD can occur in children and current data suggest that pediatric-onset NMOSD accounts for 3–5% of all NMOSD cases, depending on the diagnostic criteria and the inclusion of AQP4-antibody testing (12, 13). Median age of onset in a Chinese study was 14 (range 7–17 years) (14).

Demographic data from pediatric patients with NMOSD selected from different multinational publications are summarized in Table 1. These studies have focused primarily on AQP4-IgG related disease (6, 15–21). Overall, findings have been variable, but most studies suggest that NMOSD is more common in girls than boys, with F:M ratios ranging from 9:1 (UK) (18) to 1:1 (multinational) (19). One study from the US has noted variations in F:M ratio in different age groups, with children older than 11 years of age demonstrating a 3.5:1 ratio, and those younger than 11 a F:M ratio of 1.5:1 (6). Incidence and prevalence of pediatric-onset NMOSD are not well-characterized, but population-based studies from Europe, Asia, and South America suggest that the overall incidence of NMOSD in children and adults ranges from 0.05 to 4/100,000 per year, and the prevalence from 0.52 to 4.4/100,000 (22). In Japan, the incidence of pediatric NMOSD was reported to be 0.06 per 100,000 children (23).

**Table 1:** Demographic data of pediatric NMOSD from eight published series.

**Pediatric NMOSD population**	**German**	**French**	**Brazilian**	**UK**	**US network**	**Multinational**	**Catalonia (Spain)**	**US**
	***n*** **=** **7** **(****15****)**	***n*** **=** **12** **(****16****)**	***n*** **=** **29** **(****17****)**	***n*** **=** **20** **(****18****)**	***n*** **=** **38** **(****6****)**	***n*** **=** **12 NMO, 33 limited forms** **(****19****)**	***n*** **=** **5** **(****20****)**	***n*** **=** **16** **(****21****)**
Mean age at onset	9.6 (5–14)	14.5 (4–17.9)	13 (5–17)	10.5 (2.9–16.8)	10.2 ± 4.7	9 (0.75–17)	n/a	12 (1–15)
Female/Male ratio	2.5:1	3:1	2.6–1	9:1	<11 years = 1.5:1 >11 years = 3.25:1	1:1	1.5:1	3:1
AQP4-IgG positive	2/7	8/12	22/27	12/20	24/37	5/45	2/5	70%
MOG-IgG positive	–	–	–	–	–	25/45	1/5	–
Race	White = 6/7 Arabian = 1/7	White = 8/12 Asian = 2/12 Black = 1/12 American Indian 1/12	Caucasian = 41% Mixed = 52% African = 7%	n/a	White = 37% African American = 37% Asian = 11%	Caucasian = 91% Non-Caucasian = 9%	n/a	White = 31%
Ethnicity					Non-Hispanic/non-Latino = 37% Hispanic/ Latino = 13%			

In a retrospective study of the Catalonian population in Spain prevalence and incidence in children with NMOSD were 0.22/100,000 and 0.037/100,000, respectively. Of the 5 incident pediatric cases identified in this study, 2 were AQP4-IgG positive, 1 was MOG-IgG positive, and 2 were double seronegative (20). Further work is needed to clarify the incidence and prevalence of ***MOG-IgG related NMOSD in*** ***children***.

### Clinical Manifestations

A recent study compared demographic and clinical characteristics of pediatric and adult patients with confirmed clinical diagnoses of NMOSD and found that pediatric patients were more likely to be evaluated by an ophthalmologist (88 vs. 21%; *p* < 0.001) and rheumatologist (44 vs. 7%; < 0.001) within 6 months prior to or 6 months after their first clinical event. Children were also more likely to require hospitalization (94 vs. 55%; *p* < 0.01) and to undergo MRI of the orbits (44 vs. 11%; *p* < 0.01) within ± 30 days of their diagnosis (21). Notably, MOG-IgG antibodies are of great importance in NMOSD: in one study, 40% of individuals presenting with optic neuritis and transverse myelitis who were negative for AQP4-antibodies were found to be positive for MOG-IgG (24). In this section, we will describe features associated with either/both MOG-IgG and AQP4-IgG in children and specifically indicate which antibody these clinical features have been associated with.

#### Optic Neuritis and Transverse Myelitis

A first clinical event of optic neuritis (ON) occurred in 50–75% of patients and transverse myelitis in 30–50%, either alone or in combination according to different pediatric case series of NMOSD (6, 13, 18, 25). The frequency of AQP4-IgG seropositivity is much lower in pediatric-onset NMOSD compared with adults. In a pediatric cohort from UK, 12/24 (50%) of NMOSD patients had MOG- antibodies, while only 2 children had AQP4-IgG (26). In a recent study, 110 MOG-IgG positive patients with optic neuritis were evaluated comparing clinical characteristics and outcome according to the age of presentation: pediatric, young (18–46 years) and middle-aged (>46 years) adult patients (27). Overall, children showed better recovery of visual acuity, lower annual relapse rate, and more intracranial optic nerve involvement than the young and middle-aged groups (27). Two subsets of relapsing optic neuritis, one with discrete acute attacks named RION (recurrent isolated optic neuritis), and the other characterized by chronic relapsing inflammatory optic neuropathy with corticosteroid dependence, named CRION, seem to be associated with MOG-IgG antibodies (28) with high seropositivity rates: 7/7 children (29), 11/12 adults (30).

#### Area Postrema Syndrome

Area postrema syndrome is a core criterion of NMOSD diagnosis. Prolonged and intractable vomiting and hiccups may be the initial presentation of AQP4-IgG related NMOSD in adult and pediatric patients. Indeed, presentation with an isolated area postrema syndrome is more specific for AQP4-IgG positive NMOSD than longitudinally extensive spinal cord lesions extending to this area (31).

#### Acute Brainstem Syndromes

Diplopia, facial palsy, hearing loss, hypogeusia, pruritus, trigeminal neuralgia, vestibular ataxia, and dysarthria may be observed at similar frequencies in children (40%) and adult (33–39%) patients with AQP4-IgG seropositive NMOSD in the early stages of the disease (32, 33). Neurogenic respiratory dysfunction due to medullary respiratory center dysfunction is a possible brainstem symptom. Oscillopsia and different types of nystagmus and eye movement abnormalities may occur, due to involvement of eye movement nuclei and tracts within the brainstem.

#### Cerebral Syndromes

Hemiparesis, visual field involvement, and signs of encephalopathy may be associated with large cerebral hemispheric lesions in 16–32% of AQP4-seropositive children (6, 18). In a recently published pediatric cohort of 116 MOG-IgG seropositive patients, 4/6 children with an NMOSD phenotype had associated brain MRI findings, and 68 additional patients showed an encephalopathic presentation, including 64 with acute disseminated encephalomyelitis (ADEM) features and 22 satisfying criteria for autoimmune encephalitis without ADEM features (34).

#### Diencephalic Clinical Syndrome

The syndrome of inappropriate antidiuretic hormone secretion (SIADH) is a common presentation of AQP4-IgG related NMOSD (35, 36). Hypotension, hypersomnia, hypothermia, behavioral changes, amenorrhea galactorrhea syndrome, or narcolepsy, with anterior thalamic and hypothalamic MRI involvement should prompt a serum test for AQP4-IgG. This syndrome has been reported in a 12-year-old girl with AQP4-IgG positive NMOSD 3 months after presenting with paraparesis and lower cranial nerve dysfunction (19).

#### Coexisting Autoimmune Diseases

AQP4-IgG related NMOSD may coexist with other autoimmune diseases, including organ-specific disorders (e.g., myasthenia gravis, thyroid disease, celiac disease) and non-organ-specific disorders (systemic lupus erythematosus, Sjögren syndrome). Five pediatric patients exhibiting coexisting NMOSD and Sjögren syndrome have recently been described (37, 38). NMOSD may also coexist or be followed by NMDA-receptor antibody encephalitis or other autoimmune encephalitis (39–41).

### Differential Diagnosis

Vision loss and severe myelopathy can be observed in patients suffering from a variety of disorders and may include metabolic, ischemic, infectious/post-infectious and other inflammatory disorders. One **metabolic** etiology that may be mistaken for NMOSD includes late-onset biotinidase deficiency. It is a treatable disorder that presents distinctly from the classical form of biotinidase deficiency (42). This challenging diagnosis has been reported in adult (43) and pediatric patients (44–46). Spinal cord infarct may present with sudden, painful acute and rapidly evolving weakness and sensory abnormalities. The clinical picture may therefore be difficult to distinguish from inflammatory spinal cord disease: in one series, 41/226 adult patients who received an initial diagnosis of idiopathic transverse myelitis were eventually diagnosed with a vascular myelopathy (47). Imaging is of utility, and will show pencillike hyperintensitities (100%) or a classical “owl eye” appearance (37.5%) in anterior spinal artery infarcts (48). Evidence for spinal growth dystrophy may be present in children with spinal cord infarct (49). **Inflammatory, post-infectious and systemic inflammatory** etiologies include entities such as sarcoidosis, flaccid myelitis, and SLE. Sarcoidosis can cause both optic neuritis and longitudinally extensive myelitis (50). The predilection for a posterior-dorsal subpial gadolinium enhancement in spinal cord sarcoidosis (“trident sign”) may help distinguish this entity from NMOSD (51). Acute flaccid myelitis has been increasingly recognized in children presenting with rapidly progressive, asymmetric, and flaccid weakness frequently associated with enterovirus D68 (52–54). The syndrome is usually a pure motor syndrome. On MRI, brain imaging demonstrates dorsal pons involvement in many cases, and spinal cord involvement follows a predominantly gray matter (anterior horn cell) pattern. Optic nerve involvement does not occur in this entity (52, 53).

Systemic Lupus Erythematosus (SLE) may present with longitudinally extensive transverse myelitis which may co-occur with MS and with AQP4-IgG positive NMOSD or be a sign of lupus myelitis. Lupus Myelitis may be difficult to differentiate from SLE with NMOSD myelitis, thus evaluation of serum AQP4-IgG is essential in patients with lupus related myelitis (55). The area postrema syndrome, while suggestive of AQP4-IgG related NMOSD, may exist in other entities in children: an area postrema syndrome with intractable nausea and vomiting was recently described as the presenting symptoms of MS in a 10 year-old girl. MRI, together with laboratory testing and careful clinical evaluation, can provide important and conclusive information regarding diagnosis in cases like this (56).

### Neuroimaging in AQP4-IgG and MOG-IgG Related NMOSD

Previous literature has documented the radiographic/MRI features associated with AQP4- IgG positive NMOSD, including longitudinally extensive transverse myelitis with central/gray matter predominance in the presence of hypothalamic and periventricular brainstem lesions, namely dorsal brainstem/area postrema lesions (5). Brain abnormalities are frequently seen and may also include lesions of the corpus callosum (12–40%)—located next to the lateral ventricles with unique features such as complete involvement of the splenium and extension into the cerebral hemispheres—and tumefactive lesions of the brain. Optic nerve involvement includes the presence of longitudinally extensive T2-weighted bright and T1-weighted gadolinium-enhancing lesions of the optic nerve, often bilateral (57). These lesions may be associated with increased diffusivity, suggesting the presence of vasogenic edema. Of note, MRI features of spinal cord lesions may change with time, with longitudinally extensive lesions taking on a patchy appearance or longitudinally extensive spinal cord atrophy at follow up (58).

These features, while associated with AQP4-IgG NMOSD in the adult population, do not distinguish AQP4-IgG positive NMOSD in children from those suffering from other neuroinflammatory disorders, however. LETM is a frequent occurrence across all pediatric neuroinflammatory conditions and is almost universally seen (88%) in children with monophasic transverse myelitis (59). Importantly, recent work on MOG antibody-associated syndromes suggests that this antibody is a more frequent association with LETM in children: one series reported that 11% of children presenting with NMO or limited forms (brainstem syndrome, bilateral ON, recurrent optic neuritis, LETM) were positive for the AQP4-antibody, while 58% were MOG-IgG positive (19). Conversely, in the adult population, over half of LETM patients are AQP4-IgG positive (60, 61).

MRI features of MOG-IgG -associated disease in children include brain lesions in the majority (67%), with subcortical white matter and brainstem lesions (62), which are noted to be large with indistinct borders (63), with complete resolution in over half (53%). Conflicting reports about corpus callosum involvement exist (64). Lesions of the cerebellar peduncle distinguish children with MOG-antibodies from those with AQP4-IgG (65). As for spinal cord lesions, one small study suggests that about half of children with MOG-antibodies may have spinal cord lesions, most of which are localized to the cervical spine (62, 66). Finally, children presenting with ON in the context of MOG-IgG positivity may present with involvement of any part of the optic nerve, but are more likely to have intracranial ON involvement than those who are seronegative (51.2 vs. 17.4%, *p* = 0.009) (67).

### Visual Imaging in Pediatric MS and MOG-IgG and AQP4-IgG Related NMOSD: Optical Coherence Tomography

Imaging of the anterior visual pathway using optical coherence tomography may be of utility in distinguishing children with NMOSD from others. In an older study, children with a history of CRION, an entity now known to be highly associated with MOG-IgG antibodies (28), were found to have a marked decrease in Average Retinal Nerve Fiber Layer Thickness (RNFLT) in comparison to MS patients (50 ± 2 microns in CRION vs. 83 ± 12 in MS ON affected eyes and 107 ± 12 in healthy controls), suggesting more severe injury of the optic nerve in this entity than MS (68). More recently, in a cross sectional study, Chen et al. compared children with new onset ON who were AQP4- IgG positive, MOG-IgG positive, or negative for both antibodies. Average RNFLT in affected eyes was similar between the MOG-IgG positive (58.03 ± 8.73 microns) and AQP4-IgG positive patients (64.34 ± 12.88 microns): those who were antibody positive differed from those who were antibody negative (69). By contrast, another group found almost normal RNFLT in AQP4-IgG positive patients, with similar results to the study above otherwise-−58 microns (range 34,97; IQR 23) in MOG-IgG-positive and 82.5 microns (range 32,116; IQR 36) in seronegative patients (70). In another Chinese study of 48 pediatric patients with acute ON, children seropositive for MOG-IgG had thicker peripapillary retinal nerve fiber layers overall and in the superior and inferior quadrants than patients seropositive for AQP4 -IgG (*p* = 0.005, *p* = 0.002l, and *p* = 0.024, respectively) (67).

### Chronic Treatment Strategies: Relapse Prevention

AQP4-IgG associated NMOSD is a relapsing disorder with recurrent attacks of optic neuritis and myelitis if untreated, leading to significant morbidity from relapse-related disability (8, 71) AQP4-IgG seropositive patients have a greater than 50% risk of recurrence in the 12 months after diagnosis. AQP4-IgG seronegative patients likely represent individuals with both monophasic and relapsing inflammatory CNS disorders (72), including those with positive MOG-antibodies.

The clinical course of MOG-IgG related disease—including NMOSD and other clinical phenotypes – is less clear; although initially reported to be typically monophasic, cumulative experience now suggests that, depending on published series, 25% to almost 75% will relapse, and some data suggests that this is particularly true if the serum MOG-IgG persists (73).

Relapse prevention therapies are indicated to decrease the risk and severity of relapses and subsequent disability in patients with high risk of relapse, such as those with AQP4-IgG positive disease, in addition to patients with recurrent disease and persistent MOG- antibodies.

The current available treatment options, including the off-label use of Intravenous Immunoglobulin (IVIG), rituximab, mycophenolate mofetil, and azathioprine will be reviewed with special focus in their pediatric use. As the use of these agents has been reported in both AQP4-IgG positive patients and, in selected cases, MOG-IgG positive children with NMOSD, we will review the use of these agents together, but will specify the population of relevance under the agent being discussed. Following this section, in Position Subsection (below), we will provide suggested treatment recommendations based on available evidence by subtype (AQP4-IgG, MOG-IgG, no identified antibody).

#### Rituximab

Rituximab is an anti-CD20 chimeric monoclonal antibody that depletes B-cells that has been in off-label use for the treatment of NMOSD for more than 10 years in adult and pediatric patients (74). Various studies on children with AQP4-IgG positive NMOSD have demonstrated reduction in the annualized relapse rate (75) or a more robust response to rituximab with almost complete cessation of relapses (76).

Its use has also been described in relation to all subtypes of MOG-IgG related disease in children. In a recently published study, no new MRI lesions or clinical relapses were observed in 10/12 children with a relapsing MOG-IgG associated disorder while B-cells were suppressed on rituximab treatment, and 4/6 children had clinical relapses occurring in the context of B-cell repopulation (77). Another retrospective series reported a decrease in ARR in MOG-IgG positive children, from 2.12 to 0.67 (*n* = 9) while using rituximab (78). Notably, response to rituximab may be different in MOG-IgG and AQP4-IgG positive patients. A recent publication comparing seropositive adult NMOSD patients on rituximab showed reemergence of B cells in the majority of relapses associated with AQP4- antibodies (92.5%; 12/13) and only 20% (2/10) of relapses in those who were positive for MOG- antibodies (79).

Because there is no standard protocol for rituximab infusions, induction dosing in pediatric patients is variable among centers. Two different protocols may be used for the starting dosing: 375 mg/m^2^/dose, weekly for 4 consecutive infusions, or 500 mg/m^2^/dose (max 1 g), 2 infusions 2 weeks apart. Monitoring of B-cell counts is recommended 2–3 weeks after the last infusion to confirm CD19 suppression, and then monthly starting at 4 months, as one study has shown repopulation to occur as early as 4 months (75). We have seen B-cell repopulation occur earlier in rare cases and the 4 months recommendation may therefore not be suitable for all children. Considering the risk of relapses with B-cell repopulation, redosing regimen depends on the availability for frequent monitoring of CD19 cells and patient age: 500 or 750 mg/m^2^/dose (1,000 mg maximum) when CD19 >1%, or every 6 months. Switching from six monthly infusions to B-cell-monitoring reduces cumulative rituximab dose without apparent loss of efficacy (80). Moreover, in contrast to total CD19+ cell detected with routine techniques, the addition of assessment of subpopulations of B cells using multicolor flow cytometry, namely CD19+ CD27+ memory B cells, have been shown to be a reliable marker that correlates with biological relapse. In this study, the authors found the reemergence of naïve B cells prior to the emergence of CD19+ CD27+ memory B cells in some cases, and that this reemergence did not correlate with total B cell emergence, allowing for a decrease in the frequency of rituximab infusions (81). Low dose rituximab regimens have been described by several groups (82–84), but notably, dosing may need to be more frequent with lower doses. In a recently published study performed in adult patients with NMOSD, similar to other previously published studies (82), recovery of CD19+ B-cells analyzed by linear regression was significantly faster in patients who received low doses of rituximab (250 mg) compared to 500 mg and higher doses (85). Infusion doses of 1,000 mg yielded the longest interval before reinfusion, with a mean of 14.7 ± 6.6 months.

Safety data associated with the use of rituximab must be considered. In one study, infusion-associated adverse events were recorded in 18/144 (12.5%) children with a wide range of autoimmune and inflammatory CNS disorders, including 20 with NMOSD (74). In addition, 11 patients (7.6%) in this series developed an infectious complication during follow-up: severe (death) in two children with autoimmune encephalitis (cytomegalovirus colitis and staphylococcal toxic shock syndrome), and cytomegalovirus retinitis, pneumonia, bronchiectasis, salmonella enteritis, mastoiditis in the remaining children (74).

The risk of developing hypogammaglobulinemia appears to increase with repeated courses and long-term use of rituximab (86). Persistent hypogammaglobuninemia following rituximab use should alert the physician to the possibility of primary immune deficiency. In one pediatric series, one-third of children with autoimmune cytopenia had persistent hypogammaglobulinemia (>12 months) after treatment with rituximab, 53% (9/17) of whom later received a diagnosis of primary immune deficiency (87). Mild to serious sinopulmonary infections, urinary tract infections, and septicemia may occur associated with hypogammaglobulinemia (88, 89). To mitigate these potential side effects, we recommend pretreatment vaccinations, close B-cell monitoring to limit cumulative rituximab dose, and use of immunoglobulin replacement therapy in cases of symptomatic hypogammaglobulinemia only with IVIG at 0.4–0.6 g/kg/months, or with subcutaneous formulations (90).

#### Mycophenolate Mofetil

Mycophenolate mofetil (MMF), a prodrug of mycophenolic acid, has shown to be effective in adult and pediatric patients with NMOSD in a retrospective study of patients who were, for the most part AQP4-IgG positive (90%) (91). The median post-MMF annualized relapse rate and Expanded Disability Status Scale (EDSS) scores were significantly decreased, with 60% of patients being relapse free. Adverse events were observed in 24% of patients, including rash, amenorrhea, herpes zoster, cystitis, hypotension, fatigue, and mild hair loss. In addition, a retrospective series showed a reduction in ARR in relapsing MOG-IgG positive pediatric patients from 1.79 to 0.52 (*n* = 15), confirming safety and possible efficacy of this agent for this disorder (78).

Recommended dosing of MMF is 500–750 mg/m^2^/dose, administered orally twice a day, with a maximum of 2,000 mg total/day. Although mycophenolic acid levels can be measured (area under the curve), its use in optimizing dose both in adults and in children with NMOSD remains to be evaluated. Considering that MMF may have a slow onset of efficacy in preventing clinical attacks, its use should initially overlap with another immune therapy, such as oral corticosteroids, for up to 6 months (92).

Slower up-titration is associated with better tolerability in children, starting at 250 mg once or twice a day, increasing the dose every 7 days until reaching the final goal dose (93).

#### Azathioprine

Azathioprine is a purine analog that interferes with DNA synthesis in proliferating cells, including T and B cells. It has been shown to be a modestly effective treatment for NMO in a large cohort of AQP4-IgG positive patients including adults and children (94). Although 89% experienced reductions in relapse rate and 61% remained relapse free at 18 months after treatment initiation, at last follow-up 46% had discontinued treatment due to side effects or because of ongoing disease activity. The therapeutic benefit of azathioprine over a 5-years period in a single child wrongly classified as pediatric MS, but with a clinical phenotype of relapsing MOG-IgG associated disorder, has been recently published (95). In another series, pediatric patients with MOG-IgG positive disease receiving azathioprine had an ARR reduction from 1.84 to 1.0 (*n* = 20) (78).

Reported side effects include nausea, elevated liver function tests, diarrhea, severe leukopenia, rash, and hypersensitivity reactions. Recommended pediatric dosing is 2–3 mg/kg/day. In the past, azathioprine was one of the most frequently used preventive treatments in NMOSD due to its wide availability, low cost, and oral route. However, the need of the chronic combination with steroids to sustain its modest efficacy has substantially reduced the use of this treatment.

#### IVIG

IVIG consists of polyclonal IgG pooled from the serum of thousands of donors, and is frequently used as an anti-inflammatory treatment in a number of neurological disorders, such as Guillain-Barré syndrome and myasthenia gravis. The mechanism of by which it exerts its anti-inflammatory effect in specific diseases is unknown and may be disease dependent: it may stem from both functional domains of the IgG molecule, the antigen binding fragment (F(ab)_2_ and the fragment crystallizable region (Fc) (96).

In a retrospective series, the use of IVIG on a 2–3 monthly basis (1 g/kg/dose) was associated with reduction in relapses in adult NMOSD patients, of whom 4/6 were positive for AQP4-IgG (the remaining 2 did not have testing results available) (97). No reports of its use for chronic prevention in pediatric AQP4-IgG related NMOSD are available.

On the other hand, in MOG-IgG related NMOSD in pediatrics, several reports of use of IVIG for relapse prevention have been published. In a retrospective case series, 12 MOG-IgG positive relapsing patients receiving monthly IVIG experienced an improvement of ARR from 2.16 to 0.51 (78) In addition, a case report of a child previously diagnosed with MS and subsequently found to be MOG-IgG positive was treated with multiple therapies with ongoing breakthrough disease, including interferon and rituximab, received IVIG as monotherapy on a monthly basis for 24 months with excellent disease control (98). Dosing used in current practice is variable and ranges from 1 to 2 g/kg, with some recommending a 2 g/kg induction dose and 1 g/kg/dose monthly thereafter as maintenance.

Several retrospective analyses of IVIG related adverse events (AE) in pediatric populations have been published, with the majority of reported AEs being mild, including fever and headache (99, 100). The extent of the mild AEs may vary from center to center, and reported numbers may be as high as almost 40% (100). Other common and more serious associated AEs include aseptic meningitis, thrombosis and anaphylaxis (96).

### Future Options

Several new therapies for NMOSD have been developed in the recent years and are currently undergoing clinical evaluation in patients with both AQP4-IgG positive and negative NMOSD (101). Notably, MOG-IgG patients, if included in these trials, were not identified, as testing for MOG-IgG was not performed on AQP4-IgG negative patients. These new agents have different mechanisms of action: Eculizumab inhibits complement C5, satralizumab and tocilizumab are monoclonal antibodies against IL-6 receptor, and inebilizumab is an anti-CD19 antibody designed to deplete B cells (Table 2). These new therapeutic monoclonal antibodies are listed in Table 3 and described in more detail below.

**Table 2:** Attack prevention treatment strategies for NMO/NMOSD.

**Categories of immune therapies**	
1- Non-cell-specific interference with DNA synthesis and interference with DNA repair (cytotoxic agents)	Cyclophosphamide
2- Cell-specific interference with DNA synthesis(anti-proliferative agents)	Azathioprine Mycophenolate mofetil
3- Depletion of B-cells	Rituximab (CD20) Ocrelizumab (CD20) Ofatumumab (CD20) Inebilizumab (CD19)
4- Different mechanisms	Tocilizumab (IL-6 receptor) Satralizumab (IL-6 receptor) Eculizumab (C5 complement)
5- MS therapies that should be avoided	IFN-beta Natalizumab Fingolimod Alemtuzumab

*Adapted from (102)*.

**Table 3:** Immunosuppressive molecules for attack prevention in NMOSD.

	**Monoclonal antibody**	**Mechanism**	**Route**	**Risk**
Rituximab	Chimeric	CD20-B cell depletion	IV	Infections; Hepatitis B reactivation; Infusion-related reaction
Eculizumab	Humanized	C5 complement inhibitor	IV	Meningococcal infection; Possible PML risk; Infusion-related reaction
Satralizumab	Humanized recycling	IL-6 receptor blocker	SC	
Tocilizumab	Humanized	IL-6 receptor blocker	SC	Cardiovascular risk; Cholesterol levels
Inebelizumab	Humanized	CD19-B cell depletion	IV	Infections; Infusion-related reaction
Ofatumumab	Fully humanized	CD20-B cell depletion	SC	Infections; Infusion-related reaction; Hepatitis B reactivation
Ocrelizumab	Humanized	CD20-B cell depletion	IV	Infections; Infusion-related reaction; Hepatitis B reactivation

#### Eculizumab

Eculizumab is a humanized monoclonal antibody that targets the complement protein C5, with consequent inhibition of the complement cascade that is responsible for forming membrane attack complex (MAC) attacking AQP4 expressing cells. Eculizumab has been recently approved by the FDA for the treatment of NMOSD in AQP4-IgG positive adult patients. This approval was based on results from a Phase 3 randomized, double-blind placebo-controlled study (PREVENT), with relapse free status in 98% of patients treated with eculizumab as an add-on to stable-dose immunosuppressive therapy, compared to 63% of patients receiving placebo (103). A clinical trial to evaluate safety in youth with NMOSD is currently underway.

While there is no published experience with eculizumab in children with NMOSD, this monoclonal antibody is approved in USA, EU, Japan and other countries as first-line therapy in atypical hemolytic uremic syndrome, for both adult and pediatric patients. In addition, a recently published study reported results of eculizumab treatment also in children with severe hemolytic uremic syndrome related to Shiga-toxin-secreting Escherichia coli infection (STEC-HUS), showing a trend toward a favorable outcome in patients with persistent complement blockade (104).

The most severe adverse event associated with eculizumab is the risk of serious infection with encapsulated organisms, as the MAC is important in the immune response to these organisms. A report from the Centers for Disease Control and prevention in the United States (CDC) identified 16 cases of meningococcal disease occurring in eculizumab-treated patients between 2008 and 2016, primarily meningococcal sepsis without meningitis (105). Another recent report described four children who developed Neisseria meningitidis serogroup B (MenB)-associated invasive meningococcal disease despite previous vaccination with a multicomponent vaccine against MenB; two of them had been previously treated with eculizumab (106). Therefore, the risk of severe and potentially fatal infection with these organisms remains a concern during eculizumab treatment. Meningococcal vaccination is mandatory prior to initiation of this therapy. Other reported adverse events with eculizumab are less severe and include headache and infusion-related reactions (101).

#### Tocilizumab

Tocilizumab is an IL-6 receptor-blocking humanized monoclonal antibody. The potential use of tocilizumab in patients with NMOSD is based on the success reported in two open label studies in adults (107, 108). In addition to the control of immunological disease activity, more than 50% of patients in these studies exhibited neuropathic pain reduction. Chronic neuropathic pain occurs frequently in adult NMOSD patients and usually requires treatment with a combination of symptomatic therapies (109).

Safety concerns described with tocilizumab treatment in adult patients with rheumatoid arthritis include the risk of cardiovascular disease and increase in cholesterol levels (101). Published experience of tocilizumab use in pediatric patients includes a 14-year-old boy suffering from NMOSD and Sjogren's syndrome and two female adolescents with AQP4 IgG positive NMOSD who relapsed during rituximab treatment (38, 110). In all of them, tocilizumab led to clinical stabilization without an increased frequency of infections, or the adverse events observed in adults. To mitigate potential risks associated with use of multiple monoclonal antibodies at once, we recommend recovery of B cells prior to initiation of tocilizumab if used after rituximab failure. Of note, both SC and IV options are available and are used in practice (111).

#### Satralizumab

Satralizumab (SA237), like tocilizumab, is a humanized recombinant monoclonal antibody targeting the IL-6 receptor with immunomodulatory potential that was designed to improve pharmacokinetics by applying an “antibody recycling technology.” In a phase three trial, 83 patients with seropositive or seronegative AQP4-IgG-NMOSD, including seven adolescents aged 13–18 years, were randomly assigned to receive satralizumab at 120 mg administered subcutaneously or placebo as add on therapy. Satralizumab significantly reduced the risk of relapses with a better response in the seropositive cohort (112). Unlike observational studies of tocilizumab, pain reduction was not seen in this trial. Preparation for a pediatric clinical trial of this agent is underway.

#### Inebilizumab

Inebilizumab (MEDI-551) is a humanized anti-CD19 monoclonal antibody, an antigen that is broadly expressed by pro-B cells, pre-B cells, mature and memory B cells and plasmablasts. Thus, compared with rituximab, inebilizumab may provide broader depletion along the B cell lineage. Importantly, CD19 is expressed on the majority of plasmablasts in the bone marrow, blood, and secondary lymphoid organs: as such, inebilizumab could remove the plasmablasts that produce the AQP4-IgG with potential benefits toward mitigating the damage from these pathogenic antibodies (101).

In a multicentre, phase 3 study, 230 adult patients with an active NMOSD were randomly allocated to 300 mg inebilizumab administered intravenously or placebo, to evaluate safety and efficacy (113). The study group included patients who were seropositive (93%) or seronegative for AQP4-IgG. Overall, treatment with inebilizumab significantly reduced the risk of clinical relapses, disability worsening, MRI lesion activity, and requirement of hospitalizations compared with placebo (113). Adverse events were common in both groups, including urinary tract infections, arthralgia, and infusion-related reactions. No cases of malignancies were reported; however, the overall follow-up safety period of 12 months was too short to assess long-term risks of opportunistic infections, secondary malignancies, or hypogammaglobulinemia. There is no published experience on the use of inebilizumab in children with NMOSD yet.

#### Ofatumumab

Ofatumumab is a fully human monoclonal antibody targeting B cells that may provide an attractive alternative to rituximab since it can deplete rituximab-resistant cells that express low levels of CD20, possibly through targeting a different epitope of CD20 (114) or by increased complement-dependent cytotoxicity (115). Following the first ofatumumab clinical trial in refractory or relapsed chronic lymphocytic leukemia in 2008, it has been evaluated for various conditions including nephrotic syndrome, refractory follicular lymphoma, and MS.

There are limited case series reporting the off-label use of ofatumumab in pediatric-onset SLE, mainly in patients with severe infusion reactions to rituximab. In a retrospective review, nine patients with SLE who were treated with ofatumumab at a median age of 16 years (11–18 years) showed clinical improvement with a good safety profile (116). Additional successful pediatric exposure to ofatumumab has been recently published in two siblings with monogenic SLE before the age of 3 years (117). There is no published experience on the use of ofatumumab in children with NMOSD yet.

## Position Subsection

### Recommendations for Routine Clinical Care

Given the rarity of NMOSD in the pediatric population, the potential for significant neurological deficits associated with this disorder and need for ongoing therapy and management, we recommend assessment and ongoing follow up of children suspected to have NMOSD at a pediatric facility by a child neurologist with training in neuroinflammatory disorders. We have outlined suggested routine baseline work up and follow up schedule and laboratory/imaging assessments on Tables 4, 5.

**Table 4:** Recommendations for routine clinical care and workup in pediatric NMOSD.

**A. Recommended baseline laboratory testing and imaging**
** a. BASELINE DIAGNOSTIC TESTS: see Table 5 (1)**
** b. BASELINE INVESTIGATION FOR ASSOCIATED AUTOIMMUNITY: See Table 5 (2)**
**B. Recommended clinical follow up laboratory testing and imaging** **a.** Complete neurological assessment at baseline and at follow up 3–6 months after presentation with routine follow up q6-12 months if asymptomatic.
** b.** Routine laboratory follow-up specific to preventative/immunosuppressive therapy used Table 5.5)
** c.** MRI brain, orbits, spine. Perform at baseline, 3 months after presentation, 6 months, 12 months then yearly thereafter, or at time of acute attack.
** d.** OCT and comprehensive visual evaluation: Complete visual battery at baseline (see in Table 5.1), 6 months after presentation if abnormalities detected at baseline then yearly follow up with visual battery thereafter. Visual battery also to be performed at the time of acute attack.
**C. Subspecialty and other consultations**
** a.** Subspecialty Consultation **i.** Ophthalmology: all patients should receive ophthalmological assessment
** ii.** Urology as needed
** iii.** Rheumatology if concomitant systemic autoimmunity
** iv.** Psychiatry as needed
** v.** PICU/Respirology as needed during relapse
** b.** Rehabilitation: Physiatry, Physical Therapy, Occupational Therapy, Speech and Language Therapy. Baseline assessment suggested with follow up as needed.
** c.** Social work as needed.
** d.** Cognitive testing: Baseline cognitive testing 6 months after acute attack and every 2 years if possible, particularly in youth with cerebral involvement.

**Table 5:** Recommendations for routine baseline and risk mitigation checklist in pediatric immunosuppression.

**1- BASELINE DIAGNOSTIC TESTS**
• Brain, orbit, and spinal cord MRI, with and without gadolinium • Visual battery as clinically indicated (High Contrast and Low Contrast Visual Acuity, Visual Evoked Potentials, Visual Fields, Optical Coherence Tomography, Color Vision) • Full blood cell count – leukocytes / platelets • Liver function tests, urea, creatinine • Cholesterol, triglycerides • Pregnancy test (in juvenile female patients) • Immunoglobulin levels • Lymphocyte subsets (CD19, CD20) • Serum complement (C3, C4) • Serum protein electrophoresis • Vitamin D serum level • AQP4-IgG test in serum (CBA) • MOG-IgG test in serum (CBA) • CSF analysis - cell count, cultures, IEF for oligoclonal bands in CSF and serum • Infection screening
a) HCV and HIV serology b) HBV serology: HBV surface antigen and core antibody c) TB tests: QuantiFERON or tuberculin skin testing and chest radiograph
**2- BASELINE INVESTIGATION FOR ASSOCIATED AUTOIMMUNITY** • Antinuclear antibodies (ANA) • Extractable nuclear antigen-antibodies (ENA) • Anti-DNA antibodies • Antithyroid antibodies and thyroid function tests (TSH, T3, Free T4, Total T4) • Celiac disease **3- RISK MITIGATION FOR INFECTIOUS EVENT AT BASELINE** • Varicella zoster virus (VZV) serology • HBV serology: surface antibody • Vaccinations: complete immunization according to age (non-live vaccines >4 weeks, live vaccines >8 weeks prior to first infusion); complete pneumococcal vaccine • Complete immunization if necessary, according to VZV and HBV serology
**4- INFUSION DAY CHECKLIST FOR RITUXIMAB/OCRELIZUMAB** • **Evaluations:**
∘ Clinical assessment to exclude active infection
∘ Body weight, body temperature
∘ Blood cell count and liver function tests
•**Pre-treatment regimen** (to reduce infusion-related reactions) a) Corticosteroids: Hydrocortisone, 1 mg/kg/dose b) Antihistamines: Diphenhydramine, 1 mg/kg/dose (Max 50 mg) c) Antipyretics: Acetaminophen, 10 mg/kg/dose **5- GENERAL ASPECTS OF MONITORING IMMUNOSUPPRESSION (specific requirements depending on the molecule)** • Clinical disease activity • Repeat neuroimaging for new baseline: Brain, orbit, and spinal cord MRI (4–6 months) • Blood
a) Full blood cell count—leukopenia, lymphopenia b) Liver enzymes c) Urea, creatinine d) Cholesterol, triglycerides e) Lymphocyte subsets f) Serum complement g) Immunoglobulin levels • Urine a) Infection;
b) Renal dysfunction • New associated autoimmunity
a) Thyroid function tests and thyroid antibodies b) Antinuclear antibodies/ENA c) Celiac disease

### Recommendations for the Initiation of Preventative/Chronic Therapies

Current available treatment options for patients with NMOSD have become increasingly complex regarding different treatment efficacy, treatment adherence, and side-effect profile, in addition to specific requirements and monitoring associated with pediatric immunosuppression. Management decisions to minimize treatment-related risks require comprehensive clinical, laboratory and MRI assessment at the time of the initial diagnostic evaluation. We recommend using a standardized checklist which includes both practical measures and suggested evaluations (see Table 4). The optimal duration of treatment in this population of children is currently unknown and should be the subject of future studies.

#### Treatment of Pediatric AQP4-IgG Seropositive NMOSD

The detection of AQP4-IgG predicts relapses of myelitis and optic neuritis in adults and children with NMOSD, with cumulative neurological disability, justifying prompt initiation of immunosuppressive therapy (74) with currently available treatment options: rituximab, mycophenolate mofetil, and azathioprine (75, 76, 91, 94), until more experience and knowledge regarding safety in children is gained about newer agents such as eculizumab, satralizumab, and tocilizumab.

#### Treatment of AQP4-IgG Seronegative NMOSD

Current treatment strategies are essentially the same as for children with AQP4-IgG seropositive NMOSD. This recommendation may change if some of the investigational agents that target AQP4-IgG pathogenic mechanisms (anti-C5; anti-IL6) prove to be effective only in seropositive patients.

#### Treatment of Pediatric NMOSD Clinical Phenotype Associated With MOG-IgG

There is no consensus regarding preventive therapy for MOG-IgG-associated disease. Although it appears to be quite corticosteroid-responsive, we recommend avoiding the long-term use of oral corticosteroids and start steroid discontinuation without additional immunosuppression in children who present with a first CNS inflammatory attack and are found to be MOG-IgG seropositive. On serial serum follow-up, more than 50% of children who were seropositive at onset became MOG-IgG seronegative (64, 65, 73, 118, 119).

Persistent high titer of serum MOG-IgG has been associated with greater risk of clinical relapse. Thus, it is justifiable to consider initiation of preventive therapies in children showing persistent MOG-IgG seropositivity after a first attack or after developing a relapsing disease. Sustained remission during treatment with IVIG and with currently available immunosuppressants such as azathioprine, mycophenolate mofetil, or rituximab has been reported but the optimal drug and duration of therapy is not yet clear (77, 78, 95, 120).

## Conclusion, Gaps, and Future Directions

Here, we have reviewed recent literature detailing the distinct clinical, imaging and laboratory features of NMOSD in pediatric patients. The high prevalence of MOG-IgG antibodies in the pediatric population with an NMOSD phenotype contrasts with the adult population, where the majority of NMOSD patients are AQP4-IgG positive. We currently face the dilemma of whether to consider MOG-IgG and AQP4-IgG associated disorders separate entities or as two entities under the umbrella of NMOSD (3, 121).

MRI, structural/functional visual testing and antibody testing are of upmost importance in making this diagnosis and dictating treatment plans. Antibody testing alone appears to provide diagnostic information for the vast majority of pediatric patients presenting with NMOSD, although future studies will clarify the utility of repeated MOG-IgG antibody testing to predict relapsing disease in children. Studies have evaluated the significance of early and transient MOG-IgG positivity vs. persistent MOG-IgG antibody positivity in indicating risk for recurrent disease: further knowledge related to need for and timing of follow up MOG-IgG antibody testing is needed (64). Access to antibody testing is of great importance, as are further studies in pediatrics delineating associations between children with antibody positive disease, treatments, and outcomes.

As demonstrated in several large studies of AQP4-IgG testing in the adult population, use of a fixed cell-based immunofluorescence assay (IFA) kit and live cell-based testing provided high and almost identical sensitivity and specificity (122). Performing live cell-based assays for MOG-IgG related disease has been limited to a small number of large centers around the world. The release of a fixed cell-based IFA kit has allowed for greater access around the world to this testing. Several related studies have examined this question and suggested relatively high sensitivity of the testing kit, but lower than live cell-based testing: in one study live cell based MOG-IgG demonstrated 96% agreement between 4 national testing centers, whereas fixed cell-based assay immunofluorescence showed 90% agreement (123–125). The high sensitivity and specificity of the testing kits, and their high diagnostic yield support a need for access to both AQP4-IgG and MOG-IgG testing in all settings in which children with NMOSD might present.

While the list of treatment options in adults with NMOSD have grown, only one randomized controlled trial of NMOSD has included children, and that trial included only children older than 12 years of age. Yet, as we have shown in this review, abundant literature suggests the presence of relapsing disease in children with NMOSD in need of treatment. Importantly, all trials in NMOSD to date have focused primarily on AQP4-IgG related disease, and trials in the adult population to date have shown the greatest benefit in those who are positive for the AQP4-IgG antibody. Notably, the majority of pediatric patients with an NMOSD phenotype have antibodies to MOG-IgG, emphasizing the urgent need for new trials evaluating therapies for this subgroup of patients. In addition to randomized clinical trials, future real-life studies with international cohorts evaluating therapy effectiveness in these children are needed.

## Author Contributions

EY and ST: drafting, conceptualization, and editing. All authors contributed to the article and approved the submitted version.

## Conflict of Interest

ST serves as a non-remunerated editorial board member of *Neurology: Neuroimmunology* & *Neuroinflammation* and *Frontiers in Neurology*. She has received speaker honoraria from Biogen-Idec Argentina, Merck Serono LATAM, Genzyme, Novartis Argentina, and Novartis Pharma Inc. She is member of the Genentech-Roche Inc. NMO Scientific Advisory Committee and Chair of the NMO Relapse Adjudication Committee, Alexion Pharmaceuticals Inc. EY receives research funding from the NMSS, CMSC, OIRM, SCN, CBMH Chase-an-Idea, SickKids Foundation, MS Scientific Foundation (Canada), NIH, McLaughlin Center, Biogen MA Inc. She has served as a scientific advisor to Alexion and Hoffman-LaRoche. She has received speakers' honoraria from Excemed and MS at the Limits.

## Appendix

### Active Members of The Guthy-Jackson Charitable Foundation International Clinical Consortium (GJCF-ICC)

Hesham Abboud, Case Western Reserve University, Cleveland, OH, United States Orhan Aktas, Institute of Neuroimmunology, Duesseldorf, Germany Raed Alroughani, Amiri Hospital, Kuwait City, Kuwait Ayse Altintas, Koc University School of Medicine, Istanbul, Turkey Lilyana Amezcua, University of Southern California, Los Angeles, CA, United States Metha Apiwattanakul, Prasat Neurological Institute, Bangkok, Thailand Nasrin Asgari, University of Southern Denmark, Odense, Denmark Brenda Banwell, The Children's Hospital of Philadelphia, University of Pennsylvania, Philadelphia, PA, United States Jeffrey Bennett, University of Colorado, Denver, CO, United States Denis Bichuetti, Universidade Federal de São Paulo, São Paulo, Brazil Terrence F. Blaschke, Stanford University [Emeritus], Stanford, CA, United States James Bowen, Swedish Neuroscience Institute, Seattle, WA, United States Alexey Boyko, Prigov's Russian Scientific Research Medical University, Moscow, Russia Alexander Brandt, University of California, Irvine, Irvine, CA, United States Simon Broadley, Griffith University, Mount Gravatt, QLD, Australia Wolfgang Brück, University Medical Center Göttingen, Göttingen, Germany Edgar Carnero Contentti, Hospital Alemán, Buenos Aires, Argentina Robert Carruthers, University of British Columbia, Vancouver, BC, Canada Tanuja Chitnis, Brigham and Women's Hospital & Massachusetts General Hospital, Boston, MA, United States Jeffrey Cohen, Cleveland Clinic, Cleveland, OH, United States Guillermo Delgado-García, Instituto Nacional de Neurologia y Neurocirugia, Mexico City, Mexico Irena Dujmovic Basuroski, UNC School of Medicine, Chapel Hill, NC, United States Nikos Evangelou, University of Nottingham, Nottingham, United Kingdom Kazuo Fujihara, Tohoku University, Sendai, Japan Andrew Goodman, University of Rochester, Rochester, NY, United States Benjamin Greenberg, University of Texas Southwestern Medical Center at Dallas, Dallas, TX, United States Yael Hacohen, Great Ormond Street Hospital / UCL, London, United Kingdom May Han, Stanford University School of Medicine, Stanford, CA, United States Joachim Havla, Ludwig-Maximilians University, Munich, Germany Kerstin Hellwig, St. Josef Hospital Bochum, Bochum, Germany Jyh Yung Hor, Penang General Hospital, George Town, Malaysia Raffaele Iorio, Institute of Neurology, Catholic University of Sacred Heart, Rome, Italy Anu Jacob, Walton Centre, Liverpool, United Kingdom Sven Jarius, UniversitatKlinikum, Heidelberg, Germany Jorge Andres Jimenez Arango, Universidad de Antioquia, Medellin, Colombia Ilana Katz Sand, Icahn School of Medicine at Mount Sinai, New York, NY, United States Ho Jin Kim, National Cancer Center, Goyang-si, South Korea Sung Min Kim, Seoul National University Hospital, Seoul, South Korea Dorlan Kimbrough, Brigham and Women's Hospital & Massachusetts General Hospital, Boston, MA, United States Najib Kissani, Neurology Department, University Hospital, Marrakech, Morocco Eric Klawiter, Massachusetts General Hospital, Harvard Medical School, Boston, MA, United States Ingo Kleiter, Marianne-Strauß-Klinik, Berg, Germany Marco Lana-Peixoto, Universidade Federal de Minas Gerais, Belo Horizonte, Brazil Maria Isabel Leite, Oxford University Hospitals, Oxford, United Kingdom Michael Levy, Massachusetts General Hospital, Boston, MA, United States Yaou Liu, Xuanwu Hospital, Capital Medical University, Beijing, China Fred Lublin, Icahn School of Medicine at Mount Sinai, New York, NY, United States Youssoufa Maiga, Teaching Hospital Gabriel Touré, Bamako, Mali Yang Mao-Draayer, University of Michigan, Neurology Department, Ann Arbor, MI, United States Romain Marignier, CHU Lyon, Lyon, France Sara Mariotto, University of Verona, Italy Marcelo Matiello, Brigham and Women's Hospital & Mass General, Boston, MA, United States Maureen Mealy, Viela Bio, Baltimore, MD, United States Esther Melamed, UT Austin, Austin, TX, United States Callene Momtazee, University of California, Los Angeles, Los Angeles, CA, United States Ichiro Nakashima, Tohoku University, Sendai, Japan Jayne Ness, Children's of Alabama - Neurology, Birmingham, AL, United States Celia Oreja-Guevara, Hospital Clínico San Carlos, Madrid, Spain Jacqueline Palace, Oxford University Hospitals, Oxford, United Kingdom Lekha Pandit, K S Hegde Medical Academy, Mangalore, India Friedemann Paul, Charité University Medicine Berlin, Berlin, Germany Sarah Planchon Pope, Cleveland Clinic, Cleveland, OH, United States Anne-Katrin Pröbstel, Neurologic Clinic and Policlinic, University Hospital Basel & Department of Biomedicine, University of Basel, Basel, Switzerland Peiqing Qian, Swedish Neuroscience Institute, Seattle, WA, United States Chao Quan, Huashan Hospital, Shanghai, China Pavle Repovic, Swedish Neuroscience Institute, Seattle, WA, United States Claire Riley, Columbia University, New York, NY, United States Marius Ringelstein, Heinrich-Heine-University, Duesselforf, Germany Dalia Rotstein, St. Michael's Hospital, Toronto, ON, Canada Klemens Ruprecht, Charité University Medicine Berlin, Berlin, Germany Maria José Sá, Centro Hospitalar São João, Porto, Portugal Albert Saiz, IDIBAPS Hospital Clinic of Barcelona, Barcelona, Spain Douglas Sato, Brain Institute (InsCer) PUCRS, Porto Alegre, Brazil Ché Serguera, MIRCen INSERM/ CEA, Paris, France Eslam Shosha, Prince Sultan Military Medical City, Riyadh, Saudi Arabia Nancy Sicotte, Cedars Sinai, Los Angeles, CA, United States Sasitorn Siritho, Mahidol University, Bangkok, Thailand Aksel Siva, Istanbul University, Cerrahpaşa School of Medicine, Istanbul, Turkey Terry J. Smith, University of Michigan, Ann Arbor, MI, United States Ibis Soto de Castillo, Hospital Universitario de Maracaibo, Maracaibo, Venezuela Silva Tenembaum, Hospital de Pediatria, Buenos-Aires, Argentina Leticia Tornes, University of Miami, Coral Gables, FL, United States Pablo Villoslada, Stanford University School of Medicine, Stanford, CA, United States Dean Wingerchuk, Mayo Clinic, Scottsdale, AZ, United States Jens Wüfel, MIAC AG, Basel, Switzerland Bassem Yamout, American University of Beirut Medical Center, Beirut, Lebanon Michael R. Yeaman, University of California, Los Angeles, Los Angeles, CA, United States E. Ann Yeh, Hospital for Sick Children, University of Toronto, Toronto, ON, Canada Scott Zamvil, University of California, San Francisco, San Francisco, CA, United States.
