# Corrigendum: Pediatric NMOSD: A Review and Position Statement on Approach to Work-Up and Diagnosis

**DOI:** 10.3389/fped.2020.642203

**Published:** 2021-02-15

**Authors:** Silvia Tenembaum, E. Ann Yeh, The Guthy-Jackson Foundation International Clinical Consortium (GJCF-ICC)

**Affiliations:** ^1^Department of Neurology, National Pediatric Hospital Dr. J. Garrahan, Buenos Aires, Argentina; ^2^Division of Neurology, Department of Pediatrics, SickKids Research Institute, The Hospital for Sick Children, University of Toronto, Toronto, ON, Canada

**Keywords:** pediatric, neuroinflammation, NMOSD, MOG, treatment, diagnosis

In the original article, there was a mistake in Table 3 as published. The administration form of satralizumab is “SC” not “IV.” The corrected Table 3 appears below.

**Table 3:** Immunosuppressive molecules for attack prevention in NMOSD.

	**Monoclonal antibody**	**Mechanism**	**Route**	**Risk**
Rituximab	Chimeric	CD20-B cell depletion	IV	Infections; Hepatitis B reactivation; Infusion-related reaction
Eculizumab	Humanized	C5 complement inhibitor	IV	Meningococcal infection; Possible PML risk; Infusion-related reaction
Satralizumab	Humanized recycling	IL-6 receptor blocker	SC	
Tocilizumab	Humanized	IL-6 receptor blocker	SC	Cardiovascular risk; Cholesterol levels
Inebelizumab	Humanized	CD19-B cell depletion	IV	Infections; Infusion-related reaction
Ofatumumab	Fully humanized	CD20-B cell depletion	SC	Infections; Infusion-related reaction; Hepatitis B reactivation
Ocrelizumab	Humanized	CD20-B cell depletion	IV	Infections; Infusion-related reaction; Hepatitis B reactivation

The authors apologize for this error and state that this does not change the scientific conclusions of the article in any way. The original article has been updated.

